# Cost-effectiveness of cryptococcal antigen screening at CD4 counts of 101–200 cells/µL in Botswana

**DOI:** 10.12688/wellcomeopenres.16624.1

**Published:** 2021-03-10

**Authors:** Mark W. Tenforde, Charles Muthoga, Ponego Ponatshego, Julia Ngidi, Madisa Mine, Greg Greene, Alexander Jordan, Tom Chiller, Bruce A. Larson, Joseph N. Jarvis

**Affiliations:** 1 Department of Medicine, University of Washington School of Medicine, Seattle, WA, 98195, USA; 2 Department of Epidemiology, University of Washington School of Public Health, Seattle, WA, 98195, USA; 3 Botswana-UPenn Partnership, Gaborone, Botswana; 4 Botswana Harvard AIDS Institute Partnership, Gaborone, Botswana; 5 National Health Laboratory, Gaborone, Botswana; 6 Centers for Disease Control and Prevention, Atlanta, GA, 30333, USA; 7 Department of Global Health, Boston University School of Medicine, Boston, MA, 02118, USA; 8 London School of Hygiene & Tropical Medicine, London, UK

**Keywords:** Cryptococcal antigen, CrAg, fluconazole, pre-emptive treatment, cryptococcal meningitis, HIV, AIDS, cost-effectiveness, modelling, Botswana

## Abstract

**Background: **Cryptococcal antigen (CrAg) screening in individuals with advanced HIV reduces cryptococcal meningitis (CM) cases and deaths. The World Health Organization recently recommended increasing screening thresholds from CD4 ≤100 cells/µL to ≤200 cells/µL. CrAg screening at CD4 ≤100 cells/µL is cost-effective; however, the cost-effectiveness of screening patients with CD4 101–200 cells/µL requires evaluation.

**Methods: **Using a decision analytic model with Botswana-specific cost and clinical estimates, we evaluated CrAg screening and treatment among individuals with CD4 counts of 101–200 cells/µL. We estimated the number of CM cases and deaths nationally and treatment costs without screening. For screening we modeled the number of CrAg tests performed, number of CrAg-positive patients identified, proportion started on pre-emptive fluconazole, CM cases and deaths. Screening and treatment costs were estimated and cost per death averted or disability-adjusted life year (DALY) saved compared with no screening.

**Results: **Without screening, we estimated 142 CM cases and 85 deaths annually among individuals with CD4 101–200 cells/µL, with treatment costs of $368,982. With CrAg screening, an estimated 33,036 CrAg tests are performed, and 48 deaths avoided (1,017 DALYs saved).  While CrAg screening costs an additional $155,601, overall treatment costs fall by $39,600 (preemptive and hospital-based CM treatment), yielding a net increase of $116,001. Compared to no screening, high coverage of CrAg screening and pre-emptive treatment for CrAg-positive individuals in this population avoids one death for $2440 and $114 per DALY saved. In sensitivity analyses assuming a higher proportion of antiretroviral therapy (ART)-naïve patients (75% versus 15%), cost per death averted was $1472; $69 per DALY saved.

**Conclusions: **CrAg screening for individuals with CD4 101–200 cells/µL was estimated to have a modest impact, involve additional costs, and be less cost-effective than screening populations with CD4 counts ≤100 cells/µL. Additional CrAg screening costs must be considered against other health system priorities.

## Introduction

The 2018 World Health Organization (WHO) guidelines conditionally recommended increasing the CD4 count threshold for cryptococcal antigen (CrAg) screening and targeted fluconazole treatment from ≤100 cells/µL to ≤200 cells/µL for the prevention of cryptococcal meningitis (CM) ^1^. Patients with CD4 counts of 101–200 cells/µL are also relatively immunocompromised and at risk for CM ^2^, but prevalence of CrAg positivity in this population, estimated at 2.0% (95% confidence interval (CI): 1.2-2.7%; 21 studies) ^3^ is substantially lower than prevalence among patients with CD4 ≤100 cells/µL. The impact and cost-effectiveness of increasing the CrAg screening CD4 count threshold have not been systematically evaluated, and a better understanding of the potential impact (in terms of CM cases and deaths avoided), screening program resource needs, and cost effectiveness will inform countries as they consider changes to national screening guidelines.

 Botswana had a 2018 estimated adult HIV prevalence of just over 20%, with approximately 350,000 adults currently living with HIV ^4^. Reflex CrAg screening was adopted in national HIV guidelines in 2016 ^5^. Screening is currently reflexively performed in blood samples sent for CD4 testing and found to have a CD4 ≤100 cells/µL at the Botswana-Harvard HIV Reference Laboratory, which performs most CD4 testing for the greater Gaborone region. We previously published a cost-effectiveness analysis of CrAg screening at a CD4 threshold of ≤100 cells/µL in Botswana. Screening in this population was highly cost-effective (either cost-neutral or cost-saving across different model assumptions) and prevented a significant proportion of CM cases and deaths ^6^.

Using data and estimates from Botswana in patients with a CD4 count of 101–200 cells/µL, the objective of this analysis is to expand our CrAg screening models to include those with a CD4 count of 101–200 cells/µL, with an aim of informing policy regarding CrAg screening for patients with higher CD4 counts. As in our previous analysis ^6^, we evaluated CrAg screening among patients who are antiretroviral therapy (ART)-naïve (those targeted for pre-emptive treatment in guidelines) as well as ART-experienced patients found to be CrAg-positive through reflex CrAg screening. This ART-experienced population re-engaging in care and treatment now makes up about half of those with incident CM ^7– 9^ in the region and are likely to derive clinical benefit from pre-emptive fluconazole treatment for the prevention of CM.

## Methods

### Overview

We used a decision analytic model to evaluate the number of patients receiving CD4 testing in Botswana who are at risk of cryptococcal meningitis and (1) develop CM without CrAg screening and (2) with national reflex CrAg screening adoption, as previously described ^6^, but in this analysis focused on those with a CD4 count of 101–200 cells/µL. Briefly, CD4 count distribution data were obtained from the Botswana-Harvard HIV Reference Laboratory ^10^, and local CrAg prevalence and titre data used to predict risk for progression to CM in the CD4 101–200 cells/µL population. Local data were obtained from a 2018–2019 CrAg screening cohort of patients with advanced HIV disease in Gaborone, which included over 900 patients with a CD4 count of 101–200 cells/µL who received reflex CrAg screening and were followed for up to 6 months for incident CM and mortality ^11^. In our model, based on local estimates we assume that 650,000 CD4 tests are performed annually for the adult HIV-positive population of 350,000 (around two tests per patient) ^10^.

### Screening module

The screening module (see Figure S1 in extended data ^12^), adapted from our previous model ^6^, estimates the proportion of patients who receive CD4 testing with a CD4 101–200 cells/µL, how many of these patients receive reflex CrAg screening, the proportion who are CrAg-positive, and the proportion previously initiated on ART, i.e. “ART-experienced” (see Figure S1 in extended data and key parameter assumptions in Table 1). From country data ^10^, 5.35% of all CD4 tests performed in greater Gaborone have a CD4 T-cell count between 101 and 200 cells/µL ( Table 1). Only a small proportion (15%) of patients with a CD4 101–200 cells/µL were ART-naïve in 2018–2019. Patients were considered ART-experienced if they had a prior history of HIV viral load testing documented in the national electronic medical record, as viral load testing is exclusively performed after initiation of ART as per national guidelines ^5^. In the absence of prior documented viral load testing, a patient was assumed to be ART-naïve.

**Table 1.** Key parameters, estimates, and sources of data for base model.

	Screening Module	
Parameter	CD4 101–200 cells/µl	Source(s)
% within CD4 strata	5.35%	BHHRL data
CrAg prevalence within CD4 strata (outpatient), %	3.1%	3, 11
***Among CrAg-positive individuals:***		
Proportion with prior CM, %	35%	11
Proportion with CrAg titre ≥1:160, %	20%	11
Proportion ART-naïve, %	15%	11
Return to clinic for pre-emptive treatment, %	90%	Assumption
	Treatment Module	
Parameter	CD4 101–200 cells/µl	Source(s)
Hospitalized if missed CrAg+ and develops CM, %	80%	Assumption
10-week CM mortality	50%	8
CM relapse	17%	8
Fail pre-emptive therapy (if receive fluconazole) - High CrAg titre, ART-naive *	20%	13, 15
Fail pre-emptive therapy (if receive fluconazole) - Low CrAg titre ART-naive *	5%	13, 15
Hospitalized if fail pre-emptive therapy and develop CM	90%	Assumption
10-week mortality	25%	13
CM relapse	17%	8
Hospitalized if diagnosed with CM at urgent follow-up visit	100%	Assumption
10-week mortality	25%	13
CM relapse	17%	8

ART = antiretroviral therapy; BHHRL = Botswana-Harvard HIV Reference Laboratory; CrAg = cryptococcal antigen; CM = cryptococcal meningitis * Assumptions about failed pre-emptive therapy for ART-experienced as included in extended data and underlying data ^12^

Based on data from the prospective 2018–2019 CrAg screening cohort ^11^, among screened outpatients in the 101–200 cells/µL CD4 T-cell count range, CrAg prevalence was estimated at 3.1%, 35% of whom had a history of treated CM; thus 2.0% of screened outpatients with a CD4 count of 101–200 cells/µL are estimated to be incident CrAg positives (no history of prior CM) and the target population for pre-emptive fluconazole treatment. 

 We used serum CrAg titre data to stratify the risk of CrAg-positive patients progressing to CM ^13^, with a titre >1:160 corresponding with a high risk for incident cryptococcal disease. Approximately 20% of CrAg-positive outpatients with a CD4 101–200 cells/µL had a high CrAg titre, compared to 59% among those with lower CD4 counts of ≤100 cells/µL ^6^. For our CD4 101–200 cells/µL models, we assume that patients who screen CrAg-positive and return to clinic are started on pre-emptive fluconazole therapy and none receive a diagnostic lumbar puncture to evaluate for central nervous system infection, given the lower distribution of CrAg titres in the CD4 101–200 cells/µL population compared to ≤100 cells/µL and frequent lumbar puncture refusal in routine-care settings ^14^.

 Our base model assumes that 5% of patients with CD4 101–200 cells/µL do not receive CrAg screening due to laboratory error or assay stockout and that 10% of patients who screen CrAg-positive do not return to clinic to begin pre-emptive fluconazole, putting them at higher risk for progression to CM. Given the comparatively lower CrAg titre distribution in patients with higher CD4 counts, we expect a longer delay until progression to CM in the absence of pre-emptive fluconazole meaning that fewer patients who delay returning for follow-up appointments would be expected to develop early CM in this group than among those with CD4 cell counts ≤100 cells/µL (Figure S1 in extended data ^12^).

### Base model: CrAg screening at CD4 101–200 cells/µL, treatment for both ART-naïve and ART-experienced

The base model treatment module (see Figure S2 in extended data ^12^ and key parameter assumptions in Table 1) includes outcomes for patients (1) with a CD4 count of 101–200 cells/µL who do not receive CrAg screening, (2) who are screened and CrAg-positive but do not receive follow-up to initiate pre-emptive therapy, and (3) who are screened and started on pre-emptive fluconazole therapy.

Full modeling assumptions are detailed in a Microsoft Excel file accessible online ^12^. Risk of progression to CM is dictated by whether a patient has a high- (>1:160) or low (≤1:160) CrAg titre ^13^. Outcomes of patients who develop incident CM are informed by local mortality data from Botswana, with approximately 50% of patients dying within 10 weeks of CM diagnosis under routine care conditions ^8, 16^. Patients who are recognized as CrAg-positive and started on pre-emptive fluconazole but subsequent fail therapy and are admitted to the hospital for the management of CM are assumed to have better clinical outcomes (25% versus 50% 10-week mortality) based on limited data from South Africa ^13^. Some patients who develop CM and survive hospitalization may develop relapsed CM. Given the small proportion of these patients and small clinical and public health impact, we do not consider them further in our models.

 With reflex CrAg screening, patients receive CrAg screening based on CD4 count regardless of prior ART status. However, most (85%) patients with a CD4 count of 101–200 cells/µL are now ART-experienced according to recent cohort data from Botswana 2018–2019 ^11^. Very little outcome data exist in this disparate sub-population, which consists of patients: (1) recently started on ART; (2) ART-experienced who defaulted and are now re-establishing care; and (3) ART-experienced but with treatment failure. From local 2018–2019 cohort data in Botswana, approximately 75% of these ART-experienced patients are considered to have recently started on ART (with an undetectable HIV viral load in the previous three months), 20% are on ART but with a recent unsuppressed HIV viral load signifying treatment failure, and 5% have a history of recent ART use without a recent HIV viral load signifying likely ART default ^17^. For those recently started on ART, we assumed a 33% reduction in risk of CM for those with CD4 101–200 cells/µL compared to our previous estimates for those with CD4 ≤100 cells/µL. In our base model, based on prospective cohort data ^14^, those recently started on ART with a suppressed HIV viral load have a low risk of progression to CM without pre-emptive fluconazole therapy (7%), with a greater risk in those with ART treatment failure (60%) and ART defaulters (33%). The combined risk of progression to CM for all ART-experienced patients in the CD4 101–200 cells/µL group is assumed to be 19% without pre-emptive treatment. We estimate an 87.5% reduction in risk of incident CM with pre-emptive fluconazole (factoring in a relatively low baseline CrAg titre distribution in this group) ^18^.

### CrAg screening and treatment unit costs

Costing data for CrAg screening, pre-emptive therapy, and CM treatment costs are derived using local costing data when available ( Table 2 and underlying data ^12^) ^6^. Patients who screen CrAg-positive and receive pre-emptive fluconazole are treated with fluconazole 1200 mg/day for 2 weeks, followed by 800 mg/day for 8 weeks, then 200 mg/day maintenance fluconazole for an average duration of six months pending CD4 count recovery. For patients who progress to CM, hospital bed-day costs, factoring length of hospital admission ^8^, were derived using WHO-CHOICE estimates ^19– 21^. CM treatment costs are based on two inpatient weeks of amphotericin B deoxycholate with high-dose fluconazole, intravenous fluid and electrolyte supplementation, and laboratory monitoring, followed by consolidation and maintenance fluconazole, as recommended in national treatment guidelines ^5^.

**Table 2.** Included cost estimates for CrAg screening and pre-emptive treatment and for cryptococcal meningitis treatment.

CrAg screening and pre-emptive therapy *		
Parameter	Estimate (USD)	Source(s)
CrAg LFA	$4.71	IMMY wholesale plus additional costs
Pre-emptive fluconazole 1200 mg/day x2 weeks 800 mg/day x8 weeks 200 mg/day x26 weeks	$0.51 / 200 mg tablet x 490 tablets = $247.54	CMS; proportion with treatment failure or partial adherence
Extra visit	$9.43	Assumption
Treatment Module *		
Parameter	Estimate (%)	Source(s)
Hotel costs 17-day hospital stay	$188.51 / hospital day	8, 17
Hospital drug and procedure costs Including 14 days AmBd and FLU, 2 lumbar punctures	$202.24 (survives), $151.68 (dies)	CMS; ^8^
Post-admission costs FLU consolidation/maintenance, Extra clinic visit	$226.37	CMS
Laboratory costs 2 FBC, 4 U/E, 1 ALT	$71.00	BHHRL; WHO guideline ^1^

* Underlying data includes detailed costing estimates 12 ALT = alanine aminotransferase; AmBd = amphotericin B deoxycholate; BHHRL = Botswana Harvard HIV Reference Laboratory; CM = cryptococcal meningitis; CMS = Central Medical Stores; FBC = full blood count; FLU = fluconazole; KCl = potassium choloride; Mg = magnesium supplementation; NS = normal saline; U/E = urea and electrolyte testing; WHO = World Health Organization

### Outcomes

Our model estimates the number of CM cases and CM-related deaths nationally in the population with a CD4 101–200 cells/µL without CrAg-screening and pre-emptive fluconazole therapy along with treatment costs for CM management. With implementation of CrAg screening, we then model the number of CM cases and CM-related deaths prevented in the base model (with pre-emptive fluconazole for all CrAg-positive patients) along with associated costs for screening, pre-emptive fluconazole therapy, and CM treatment. We estimate the cost per death averted and cost per disability-adjusted life year (DALY) saved compared to no screening, assuming an average age of death of 36 years ^8^. With a 3% annual discount rate and age-specific life expectancy from WHO Global Health Observatory, 21.4 DALYs are saved per avoided death ^6, 22^. 

### Sensitivity analyses

Three main sensitivity analyses are reported to account for key areas of parameter uncertainty. The complete Excel-based model is provided as underlying data ^12^ so that alternative sensitivity analyses can be completed by interested readers.

Sensitivity analysis 1 (SA1): In this analysis, we assume that in some real world settings a lower proportion of CrAg-positive patients are started on pre-emptive fluconazole after laboratory testing (50% versus 90% in the base model) because of programmatic barriers such as inadequate communication of test results to clinics, a lack of fluconazole availability in clinics, lack of provider awareness of treatment guidelines, or for other reasons. This analysis still assumes that 90% of patients attended in outpatient clinics and receiving CD4 testing will stay engaged in health care. Other parameters remain the same as the base model.

Sensitivity analysis 2 (SA2): In this model, we assume less benefit of pre-emptive fluconazole in CrAg-positive patients, with a 75% rather than 87.5% reduction in incident CM. This is to account for significant uncertainty in the benefits of pre-emptive fluconazole in this population with a higher CD4 count, and for possible sub-optimal adherence to therapy. Other parameters remain the same as the base model.

Sensitivity analysis 3 (SA3): In this model, we test our parameters with a higher proportion of ART-naïve patients receiving CD4 testing and CrAg screening (75% versus 15% in the base model). Other parameters remain the same as the base model. This is to provide estimates applicable to settings with less mature ART programmes where a higher proportion of individuals with CD4 counts of 101–200 cells/µL are likely to be ART-naïve.

## Results

### Cryptococcal meningitis cases and costs without screening

Without CrAg screening ( Table 3), we estimate 142 annual cases of incident CM in Botswana among those with a CD4 test result 101–200 cells/µL. Unlike in our prior analysis of screening in the CD4 ≤100 cells/µL sub-population, most of these incident CM cases (113 of 142, 79%) are in ART-experienced patients ^6^. Of patients with incident CM, 60% (85/142) are estimated to die (including those diagnosed and managed in hospital and those who die outside of the hospital without a confirmed diagnosis). The total estimated CM treatment costs are $368,982 annually for the health care system.

**Table 3.** Cryptococcal meningitis outcomes and costs of treatment without CrAg screening. *

Population: CD4 101–200 cells/µL	Results - ART-naïve		Results - ART- experienced		Total
	Number patients	Cost for patients (USD)	Number patients	Cost for patients (USD)	
**Identified for preemptive treatment** **(but did not receive), but did not** **develop CM – survives**	0.0	0	0.0	0	
**Identified for preemptive treatment,** **receives treatment, survives**	0.0	0	0.0	0	
**Not hospitalized, dies**	5.8	0	22.5	0	
**Hospitalized, dies < 10 weeks**	11.6	31,870	45.0	123,939	
**Hospital, survives maintenance**	9.6	36,191	37.4	140,742	
**Hospital, CM relapse**	2.0	7,413	7.7	28,827	
**Total Treatment Costs**		75,474		293,508	368,982
**Total Screening Costs (reflex policy)**		0		0	0
**Total Costs**		75,474		293,508	368,982
**Total Cases of CM**	28.9		112.6		142
**Total Deaths from CM**	17.4		67.5		85

ART = antiretroviral therapy; CrAg = cryptococcal antigen; CM = cryptococcal meningitis * Models assumes 650,000 CD4 T-cell count tests performed annually in Botswana

### Base model: CrAg screening at CD4 101–200 cells/µL, treatment for both ART-naïve and ART-experienced

With implementation of reflex CrAg screening ( Table 4), 33,036 CrAg tests are performed at a cost of $155,601. Pre-emptive treatment averted 48 deaths compared to no screening (1,017 DALYs saved). While CrAg screening costs an additional $155,601 compared to no screening, treatment costs fall by $39,600 (preemptive treatment plus hospital-based CM treatment), for a net increase of $116,001 ( Table 5). Compared to no screening, high coverage of CrAg screening and pre-emptive treatment for CrAg-positive individuals in this population is associated with a cost of $2440 per one death averted or $114 per DALY saved ( Table 5).

**Table 4.** Outcomes with CrAg screening and pre-emptive fluconazole for ART-naïve and ART-experienced (base model).

Population: CD4 101–200 cells/µL	Results - ART-naïve		Results - ART- experienced		Total
	Number patients	Cost for patients (USD)	Number patients	Cost for patients (USD)	
**Identified for preemptive treatment (but did** **not receive), but did not develop CM – survives**	6.6	0	41.9	0	
**Identified for preemptive treatment, receives** **treatment, survives**	75.3	19,361	453.3	116,472	
**Not hospitalized, dies**	2.5	28	9.9	46	
**Hospitalized, dies < 10 weeks**	5.1	14,004	20.0	55,142	
**Hospital, survives maintenance**	6.7	25,203	20.6	77,989	
**Hospital, CM relapse**	1.4	5,162	4.2	15,974	
**Total Treatment Costs**		63,759		265,623	329,382
**Total Screening Costs (reflex policy)**		155,601		0	155,601
**Total Costs**		219,360		265,623	484,983
**Total Cases of CM**	15.5		54.7		70
**Total Deaths from CM**	7.5		29.9		37

ART = antiretroviral therapy; CrAg = cryptococcal antigen; CM = cryptococcal meningitis * Models assumes 650,000 CD4 T-cell count tests performed annually in Botswana

**Table 5.** Summary of costs and outcomes for no screening and screening plus pre-emptive treatment.

Population: CD4 101–200 cells/µL	Deaths	Costs (USD)	Change in costs (USD)	Change deaths (deaths avoided)	DALYs saved	Cost per death averted (USD)	Cost per DALY saved (USD)
**No screening**	85	368,982	n/a	n/a	n/a	n/a	n/a
**Base model: Screening 101–200 ,** **preemptive treatment to both ART-** **naïve and ART-experienced**	37	484,983	116,001	48	1017	2440	114

ART = antiretroviral therapy; CrAg = cryptococcal antigen; CM = cryptococcal meningitis; DALY = disability-adjusted life year * Models assumes 650,000 CD4 T-cell count tests performed annually in Botswana

### Sensitivity analyses

**Figure 1.**
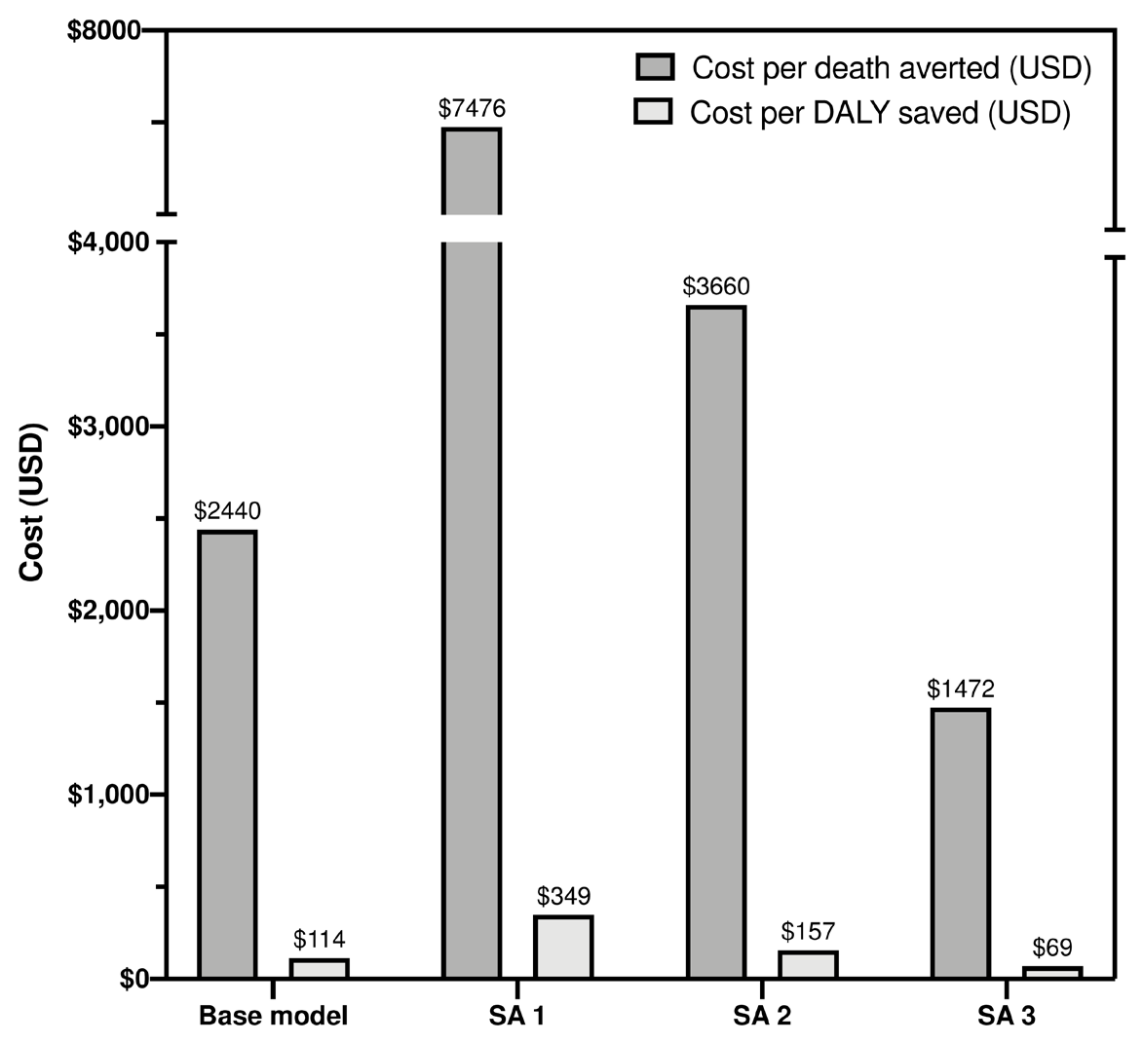
Estimated costs per death averted and disability-adjusted life year saved under base model and sensitivity analyses. DALY = disability-adjusted life year; SA = sensitivity analysis; USD = United States dollar.

**Figure 2.**
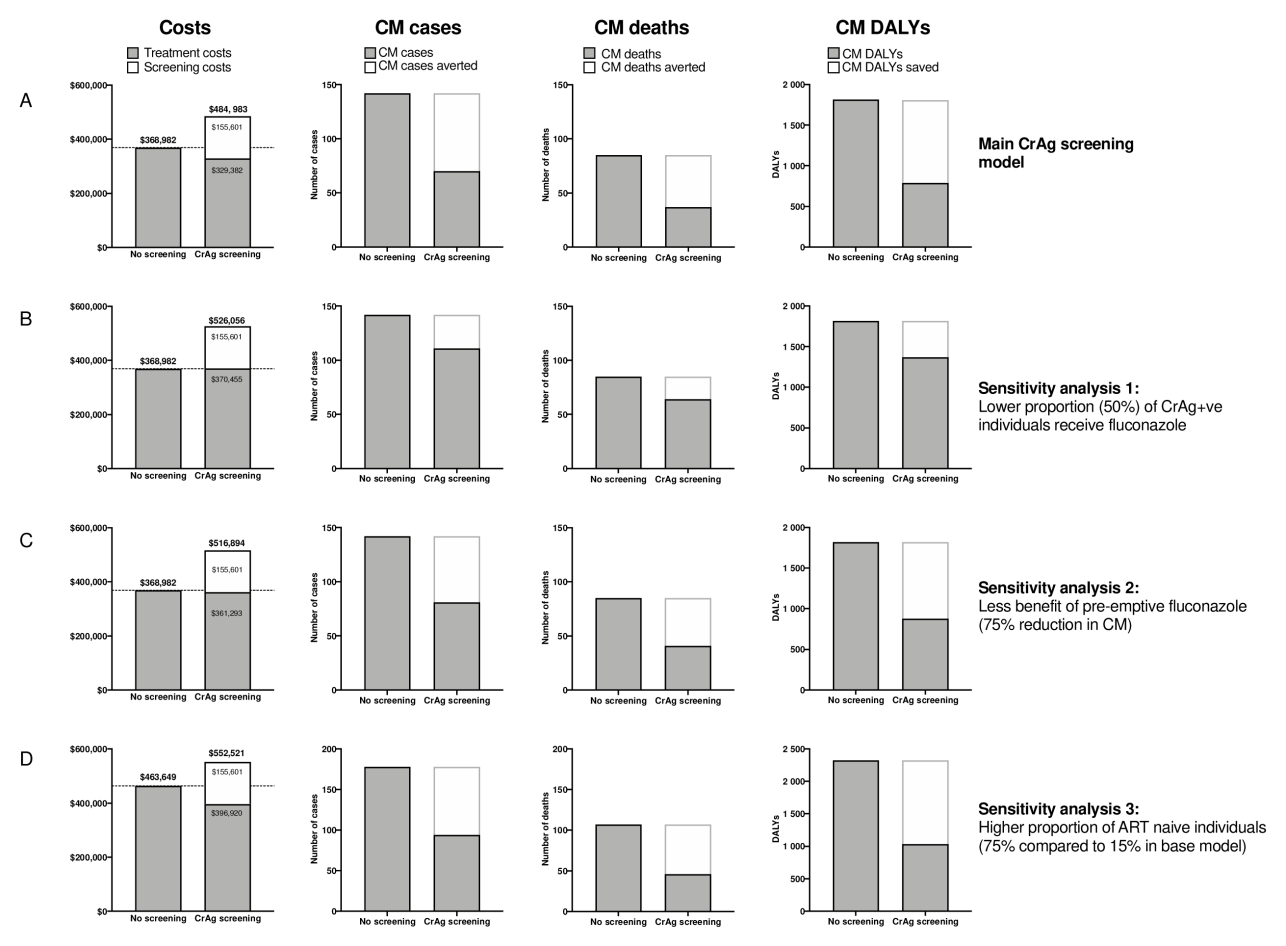
Summary of total estimated programmatic costs, number of cryptococcal meningitis cases, cost per deaths averted, and cost per disability-adjusted life year saved under base model and sensitivity analyses. ART = antiretroviral therapy; CM = cryptococcal meningitis; CrAg = cryptococcal antigen; DALY = disability-adjusted life year.

SA1 and SA2 assume a lower proportion of CrAg positive are started on pre-emptive fluconazole and a reduced benefit of pre-emptive fluconazole therapy for CrAg-positive patients with a CD4 101–200 cells/µL, respectively, which may be more realistic under many routine care conditions. Both models will therefore result in a smaller public health benefit to CrAg screening and a higher incremental cost per death or DALY saved. For SA1, an estimated 25% (21/85) of deaths are averted with treatment of both ART-naïve and ART-experienced with a cost per death averted of $7476 or $349 per DALY saved ( Figure 1). For SA2, 52% (44/85) of deaths are averted with treatment of ART-naïve and experienced at a cost per death averted of $3360 or $157 per DALY saved. For SA3, assuming a higher proportion of ART-naïve patients are among the screened population (75% versus 15%) results in slightly enhanced public health benefit and cost per death or DALY saved as the base model (see Excel file with underlying data ^12^), with an estimated 56% (60/107) reduction in CM-related deaths at a cost of $1472 per death averted and $69 per DALY saved. Overall estimated costs, number of CM cases, number of deaths averted, and DALYs saved for the base model and sensitivity analyses are summarized in Figure 2. 

## Discussion

We used local data from Botswana to estimate the cost and impact of laboratory-based CrAg screening for HIV-positive patients with CD4 counts 101–200 cells/µL across a range of assumptions. Compared to screening in patients with very advanced HIV disease (CD4 ≤100) ^6^, the benefit of screening for those with higher CD4 counts, in terms of avoided CM cases and deaths, is less marked. Under base model assumptions compared to no screening for this higher CD4 category of patients, 48 deaths are averted and screening costs of about $156,000 are offset by a $40,000 reduction in treatment costs (mainly CM-based hospital care and treatment). The cost per death averted through CrAg screening and pre-emptive fluconazole therapy was estimated at about $2400 ($114 per DALY avoided). If substantially fewer patients who screen CrAg-positive are started on pre-emptive fluconazole therapy (50% compared to 90% in the base case analysis), which might better reflect some real-world conditions without focused efforts on providing preemptive treatment, the estimated cost per death averted increases to over $7000 (and $349 per DALY saved).

 Compared to prior analyses of CrAg screening for patients with CD4 ≤100 cells/µL, fewer CrAg positive patients with CD4 101–200 cells/µL are likely to have high CrAg titres (~20% in the higher CD4 group compared to ~60% in the lower CD4 group ^11, 14^), which reduces the risk of incident CM and failure of pre-emptive fluconazole ^13^. In addition, overall CrAg prevalence among the CD4 101–200 cells/µL group is estimated to be less than the CD4 ≤100 cells/µL group. Both of these factors reduce the benefit of screening among patients with higher CD4 counts. 

 As of 2021, Botswana has an advanced ART program. Whereas CrAg screening guidelines have primarily focused on ART-naïve patients ^1^, a large majority of patients with advanced HIV disease in Botswana are ART-experienced. From recent data of 2018–2019, we found that most outpatients receiving CD4 testing in the greater Gaborone region with a CD4 count of 101–200 cells/µL were currently on ART ^11^. We included a sensitivity analysis assuming that a majority (75%) of patients who received CD4 testing and CrAg screening were ART-naïve, which may inform other health systems with a higher proportion of ART-naïve patients receiving CrAg screening with ART initiation. This sensitivity analysis showed a slightly better impact and cost-effectiveness compared to the base model assuming most patients were ART-experienced although screening was still not cost-neutral or cost-saving.

 This study is subject to a number of limitations. First, we used local clinical and costing estimates. The relative costs of CrAg screening, pre-emptive fluconazole therapy, and CM treatment between different health systems may impact cost-effectiveness of CrAg screening between settings. Secondly, the base model presents optimistic management assumptions, with about 90% of CrAg-positive patients started on pre-emptive therapy and a nearly 90% reduction in incident CM assuming relatively good adherence. Sensitivity analyses showed that under less ideal assumptions the cost per death averted or DALY saved could increase substantially. Notably, under no model assumptions was CrAg screening in this population estimated to be cost-neutral or cost-saving. Third, there is considerable uncertainty in model estimates, particularly regarding the clinical benefit of pre-emptive therapy in ART-experienced patients. Fourth, we used local CD4 testing practices in Botswana to inform these estimates. Alternative testing practices, such as testing only ART-experienced patients who have treatment failure based on HIV viral load testing or who are newly engaging in care following default, may result in greater cost-effectiveness of reflex CrAg testing.

 In summary, nationwide CrAg screening in patients with advanced HIV disease with a CD4 count of 101–200 cells/µL in Botswana is estimated to have a modest impact (48 deaths avoided annually) for a modest additional cost to the overall HIV/AIDS care and treatment program ($116,000), with a relatively low cost per DALY saved ($114 base case). With less coverage of pre-emptive treatment for CrAg positive patients, the cost per DALY saved, compared to no screening, is estimated at about $350. Overall, expanding screening to this higher CD4 count population would be estimated to require about 33,000 additional CrAg tests annually, with an estimated cost of about $156,000. The decision of whether or not to adopt CrAg screening in national HIV advanced disease guidelines among patients with higher CD4 counts (101–200 cells/µL) will rely on the availability of these additional resources and competing health system priorities.

## Data availability

The data referenced by this article are under copyright with the following copyright statement: Copyright: ï¿½ 2021 Tenforde MW et al.

Data associated with the article are available under the terms of the Creative Commons Zero "No rights reserved" data waiver (CC0 1.0 Public domain dedication). 

### Underlying Data

Open Source Framework: Cryptococcal antigen screening in Botswana, CD4 101–200. https://doi.org/10.17605/OSF.IO/GN98V ^12^.

This project contains the following underlying data:

- CD4 100–200 full model data.xlsx

### Extended data

Open Source Framework: Cryptococcal antigen screening in Botswana, CD4 101–200. https://doi.org/10.17605/OSF.IO/GN98V ^12^.

This project contains the following extended data:

- Figures S1 (flowchart of screening module) and S2 (flowchart of treatment module)

Data are available under the terms of the Creative Commons Zero "No rights reserved" data waiver (CC0 1.0 Public domain dedication).
