# Supplementary material for: Cost-effectiveness of cryptococcal antigen screening at CD4 counts of 101–200 cells/µL in Botswana
Source: Wellcome Open Res. 2021 Dec 9;6:55. Originally published 2021 Mar 10. [Version 2] doi: 10.12688/wellcomeopenres.16624.2 (PMC8767426; doi:10.12688/wellcomeopenres.16624.2)
Supplement: Peer review history

# Peer review: Reviewer response for version 2

**DOI:** 10.21956/wellcomeopenres.19327.r47575

**Published:** 2022-01-18

**Authors:** Stephanie Popping

**Affiliations:** 1 Department of Viroscience, Erasmus MC, Rotterdam, The Netherlands; 2 Department of Medical Microbiology and Infectious Disease, Erasmus MC, Rotterdam, The Netherlands

With the modifications I provide my approval for indexing.

Is the work clearly and accurately presented and does it cite the current literature?

Yes

If applicable, is the statistical analysis and its interpretation appropriate?

Yes

Are all the source data underlying the results available to ensure full reproducibility?

Yes

Is the study design appropriate and is the work technically sound?

Yes

Are the conclusions drawn adequately supported by the results?

Partly

Are sufficient details of methods and analysis provided to allow replication by others?

Yes

Reviewer Expertise:

NA

I confirm that I have read this submission and believe that I have an appropriate level of expertise to confirm that it is of an acceptable scientific standard.

# Peer review: Reviewer response for version 1

**DOI:** 10.21956/wellcomeopenres.18328.r45594

**Published:** 2021-09-22

**Authors:** Stephanie Popping

**Affiliations:** 1 Department of Viroscience, Erasmus MC, Rotterdam, The Netherlands; 2 Department of Medical Microbiology and Infectious Disease, Erasmus MC, Rotterdam, The Netherlands

This research describes a decision based model to analyse the cost-effectiveness of cryptococcal antigen screening among people living with HIV with an CD4 cell-count between 101-200 cells. 

 The authors use a previously published adapted model of which all the data is online available. The performed sensitivity analyses for several uncertainties in their data. It is an interesting topic as the WHO changed the CD4 threshold for cryptococcal screening.  

 I have some minor comments/considerations:

- It is unclear from the methods how much simulations are run in the model 
- Table 2 says "hotel costs" I think this should be hospital
- What is the willingness to pay threshold in Botswana. It is hard to make the conclusion whether screening people with a CD4 cellcount between 101-200 is cost-effective or even cost-saving if someone is not familiar with the numbers. Maybe also add the total HIV/AIDS care and treatment program budget just to place the amount in perspective
- Limitation 1 of the study can be addressed by changing this in a sensitivity analysis. That would make the work more useful for other countries too

Is the work clearly and accurately presented and does it cite the current literature?

Yes

If applicable, is the statistical analysis and its interpretation appropriate?

Yes

Are all the source data underlying the results available to ensure full reproducibility?

Yes

Is the study design appropriate and is the work technically sound?

Yes

Are the conclusions drawn adequately supported by the results?

Partly

Are sufficient details of methods and analysis provided to allow replication by others?

Yes

Reviewer Expertise:

medical microbiology, virology.

I confirm that I have read this submission and believe that I have an appropriate level of expertise to confirm that it is of an acceptable scientific standard, however I have significant reservations, as outlined above.

# Peer review: Reviewer response for version 1

**DOI:** 10.21956/wellcomeopenres.18328.r43398

**Published:** 2021-04-28

**Authors:** Elizabeth Nalintya

**Affiliations:** 1 Infectious Diseases Institute, College of Health Sciences, Makerere University, Kampala, Uganda

The study title is short and smart and gives a glimpse into what the paper is about. The study is well introduced however I would reorganize the paragraphs to get a better flow, that is:

 The first sentence of paragraph two can be moved to the beginning of the introduction to draw the picture on how big the HIV problem is in Botswana upfront and then talk about the rationale after. I would merge and reorganize the first two paragraphs.

 In the methods section, a great deal of work has gone into explaining how the different estimates were arrived at and what type of data was used to arrive at the estimates. This however blurs the description of the actual modeling. It would be beneficial to the reader to get a quick snap short of the final model (could be in a summarized figure placed within the text explaining the methods) This is especially because the figure S1 is very busy and can get confusing, the reader needs to understand what the final model is before trying to understand the smaller details of how the model was arrived at.

 The screening model clearly talks about the estimates included and how they are arrived at. The viral load(VL) test is used as the proxy for ART experience, it would be good to know if all these VL tests are done after 6 months of ART or at what time point this is done. Pragmatically with all logistical challenges in resource limited settings, a recommendation to do a six month viral load will mean the viral load was done at about  month eight or nine after ART start. Wondering if this choice of defining ART experience could have lumped many ART experience persons as non experienced. 

 Page 3, the last paragraph talks about the assumptions for those who did not receive CrAG screening, however its not clear where these estimates are derived from. Is this lab data or data from the prospective cohort. Also it seems to belong under the next subheading and yet has been placed under the screening model.

 The base model, the cost analysis and the sensitivity analysis are presented well and in detail and are supplemented by the tables giving a clear picture of what was being done. The discussion of study findings is comprehensive and puts them in context and the conclusions have been derived systematically from the data presented.

Is the work clearly and accurately presented and does it cite the current literature?

Yes

If applicable, is the statistical analysis and its interpretation appropriate?

I cannot comment. A qualified statistician is required.

Are all the source data underlying the results available to ensure full reproducibility?

Yes

Is the study design appropriate and is the work technically sound?

Yes

Are the conclusions drawn adequately supported by the results?

Yes

Are sufficient details of methods and analysis provided to allow replication by others?

Yes

Reviewer Expertise:

Cryptococcal meningitis

I confirm that I have read this submission and believe that I have an appropriate level of expertise to confirm that it is of an acceptable scientific standard.
